# Retinal Nerve Fibre Layer Thinning in Patients with Thalassaemia, Iron Deficiency Anaemia, and Anaemia of Chronic Diseases

**DOI:** 10.1155/2020/9268364

**Published:** 2020-11-18

**Authors:** Suhana Datta, Krishnapada Baidya, Madhumita Banerjee, Somnath Mahapatra, Sudeb Mukherjee

**Affiliations:** ^1^Department of Ophthalmology, Regional Institute of Ophthalmology, Medical College, Kolkata, West Bengal, India; ^2^Department of Ophthalmology, Regional Institute of Ophthalmology, Medical College, Kolkata, West Bengal, India; ^3^Department of Pathology, University College of Medical Science, New Delhi, India; ^4^Department of Cardiology, AMRI Hospital, Kolkata, West Bengal, India

## Abstract

**Purpose:**

Retinal nerve fibre layer (RNFL) is a sensitive structure, which is affected by anaemia due to hypoxia. A timely detection of RNFL thinning may aid preventing devastating complications. Optical coherence tomography (OCT) measures RNFL thinning with accuracy and helps in detecting thinning of the retinal layer in anaemic patients. This study was destined to evaluate thinning of RNFL in anaemic patients and their correlation with the haemoglobin level.

**Methods:**

It was a prospective comparative study. Total of 151 patients were included in this study. Patients with retinal diseases were excluded from this study. After initial evaluation, haematological and ophthalmological parameters were measured. RNFL was measured with OCT and corroborated with the Hb level and analysed accordingly. EPI and SPSS softwares were used for detail analysis and the correlation between RNFL thinning and the Hb level. Initially, each eye was separately assigned a value (0, 1, and 2) (normal, borderline, and abnormal, respectively) as per the severity of thinning, and then, the sum of the scores of both eyes were considered as a separate variable, and a multiple linear regression analysis was performed with the independent variables.

**Results:**

RNFL thinning was found to be significant in each group of patients. There was a strong correlation of RNFL thinning with degree of anaemia.

**Conclusions:**

Thalassaemia, iron deficiency anaemia, and anaemia of chronic diseases are associated with the significant damage to RNFL. Degree of anaemia is the most important parameter for such thinning of the RNFL layer.

## 1. Introduction

The retinal nerve fibre layer (RNFL) is formed by retinal ganglion cell axons, which collect the visual impulses that begin with the rods and cones. These impulses travel through the ganglion cells as they pass from the rods and cones to the RNFL. In the retina, the axons are spread out as a thin layer and appear as opaque striations. The retinal nerve fibre layer (also known as nerve fibre layer or stratum opticum or RNFL) is formed by the expansion of the fibres of the optic nerve. It is the thickest near the porus opticus, gradually diminishing towards ora serrata. The nerve fibre layer essentially consists of the unmyelinated axons of the ganglion cell, which converge on the optic nerve head, passes through lamina cribrosa, and become ensheathed by myelin posterior to lamina. In addition to axons of ganglion cells, this layer also contains the centrifugal nerve fibre, processes of Muller's cell, neuroglial cell, and retinal vessel. Thickness of the nerve fibre layer around different quadrants of optic disc margin progressively increases from the most lateral quadrant (thinnest) to upper temporal and lower temporal quadrants and ultimately to the most medial quadrant, which is thickest [1]. RNFL, which is a sensitive structure, can be stimulated by several processes (harmful situations) to undergo apoptosis. These factors include high intraocular pressure, high fluctuation of intraocular pressure, inflammation, vascular disease, and any kind of hypoxia which may lead to retinal nerve fibre loss leading to vision loss and loss of visual field [2].

Retina requires regular oxygen support to maintain its structural and functional integrity. Inner retinal layers, which receive their blood supply from superficial and deep capillary plexus of the central retinal artery, are more susceptible to hypoxic alterations. Retinal ischaemia, a significant component of many visual disorders, has a significant role in development and progression of eye diseases such as diabetic retinopathy, retinal vascular occlusion, glaucoma, and retinopathy of prematurity [3].

Hypoxia causes neuronal death through various mediators and mechanisms. Retinal ganglion cells are known to be damaged by a reduction in normal perfusion and oxygen saturation [4, 5].

Anaemia causes retinopathy in 28% of patients, especially when there is coexisting thrombocytopaenia (38%). As the severity of anaemia increases, the risk of retinopathy increases, particularly when the haemoglobin (Hb) level is below 6 gm/dL. A variety of pathologic changes occurring due to and associated with anaemia are implicated in the clinical features of anaemic retinopathy [6]. Anaemia causes retinal hypoxia, which leads to infarction of the nerve fibre layer and clinically manifests as cotton wool spots. Retinal hypoxia also leads to vascular dilatation, increased transmural pressure owing to hypoproteinemia, and microtraumas to the vessel walls [7].

Thalassaemia is a genetic disorder characterised by a quantitative defect in globin chain synthesis that results in an abnormal form of haemoglobin. Clinical features of thalassaemia vary from a minor trait to a major form along with its myriad complications. The requirement of blood transfusion in a major variety of thalassaemia poses a huge burden in lives of those patients. Apart from anaemic hypoxia, long term complication of blood transfusion, namely, iron overload, plays an important causative role in thinning of the RNFL, as noted from several studies [6, 8, 9].

Optical coherence tomography (OCT) provides an objective and quantitative measurement of RNFL thickness by measuring echo time delay and intensity of backscattered light from different retinal layers using a low coherence interferometry. The most recent technology, spectral domain OCT, uses a spectrometer as a detector of OCT signal [10, 11]. Spectral domain OCT (SD-OCT) has benefits over the time domain OCT (TD-OCT) such as higher axial resolution (3–6 *μ*m) up to 200 times faster (2700 axial scans/sec) scanning speed and better reproducibility [12].

Very few studies have been conducted to establish the relation of anaemia and retinal nerve fibre thickness. This study was destined to evaluate any association between these parameters and to find any correlation between anaemia and the RNFL.

## 2. Methods

This study was performed to assess the retinal nerve fibre thinning in association with hypoxia as a result of chronic anaemia (iron deficiency anaemia, thalassaemia, and chronic anaemia). This study also destined to assess and correlate RNFL changes with the haemoglobin level and to find out which fibre of retinal nerve fibres is affected the most. The study was conducted in Regional Institute of Ophthalmology, Medical College Hospital, Kolkata. Ethical clearance was taken from Institutional Ethical Committee. Patients were informed about the study in detail, and consent was taken in written format. Patients attending the outpatient department of Calcutta Medical College were included and followed up till one and half years. Patients with chronic anaemia who required blood transfusion were included in this study. They were initially evaluated in detail (all epidemiological parameters), and all necessary examinations were performed that included the haemoglobin level and categorization to different class of anaemia. Ophthalmological examination included visual chart examination, slit-lamp biomicroscopy with 90 D, applanation tonometry (to rule out raised intraocular pressure), indirect ophthalmoscopy, and finally optical coherence tomography (OCT) for assessment of retinal nerve fibre thickness. Those patients who had papilloedema, papillitis, glaucoma, retinal vascular disorders, and retinal detachment were excluded from the study.

## 3. Results

This study consisted of a total number of 151 patients who were suffering from thalassaemia major, E-beta thalassaemia, iron deficiency anaemia, and chronic anaemia. One patient was excluded due to optic disc oedema. Patients were selected on the basis of inclusion criteria, and ophthalmological examination was performed properly. OCT was performed, and RNFL was measured. This helped to assess the early detection of vision loss. The results were tabulated and analysed accordingly with the help of SPSS and EPI info softwares using the regression model.

### 3.1. Age Distribution

The mean age of this study population was 23.6 years with std. dev: 10.65, and the age ranges from 8 years to 51 years.

### 3.2. Gender Distribution

Out of total population (*n* = 150), male population was 51 and female was 99 in this study.

### 3.3. Disease Distribution

Out of total 150 patients, 64% of patients were suffering from thalassaemia. Out of this thalassaemic population, 50% of the population was of thalassaemia major and rest of the 50% was of E-beta thalassaemia.

### 3.4. Specific Diseases Distribution

**Figure 1:**
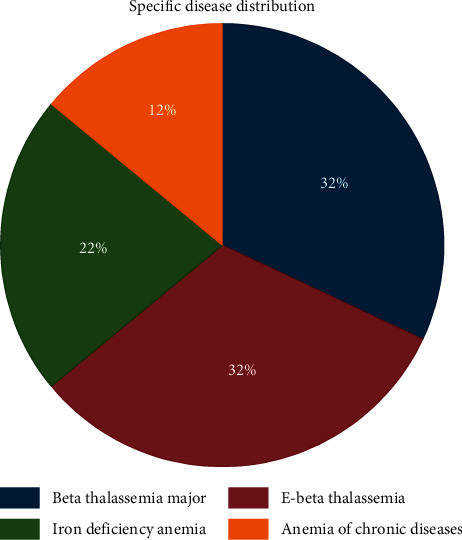
The distribution of the study population according to specific disease (*n* = 150).

Figure 1 shows the distribution of the study population according to specific diseases. 32% of the population was of thalassaemia major, 32% of the population was E-beta thalassaemia, 22% of the population was of iron deficiency anaemia, and 12% of the population was of anaemia in chronic diseases (SLE, rheumatoid arthritis, and liver disease).

### 3.5. Haemoglobin Distribution

The haemoglobin level of the study population ranged from 4 gm/dl to 10 gm/dl with a mean value of 7.50 gm/dl (std. dev: 1.16). The mean value and range of haemoglobin in each subset of anaemic population are depicted in Table 1.

### 3.6. Blood Transfusion Duration

Most of the thalassaemic patients in this study had a history of blood transfusion for long. The average number of years of blood transfusion in this study was found to be 11.4 years (nonthalassaemic cases have zero years of transfusion).

### 3.7. Chelation Therapy, Serum Ferritin, and Blood Indices

Almost all thalassaemic patients were receiving chelation therapy with defarasirox. RBC indices and the serum ferritin level are provided in Tables 1 and 2.

### 3.8. Retinal Nerve Fibre Layer (RNFL) Abnormality in the Study Population

**Figure 2:**
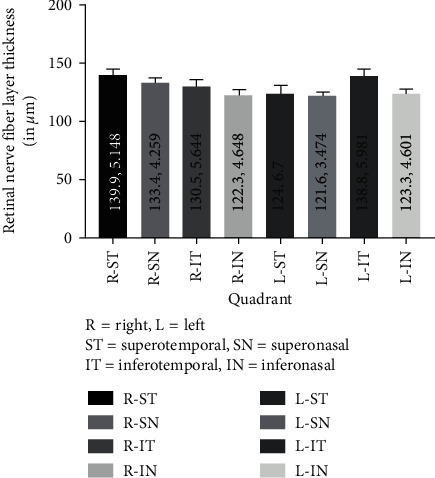
Retinal nerve fibre layer (RNFL) thickness in each quadrant altogether. Retinal nerve fibre layer thickness for each quadrant (mean, standard error of mean).

The Figure 2 shows the overall RNFL thickness in each eye and altogether as a cumulative percentage. Here, 0, 1, and 2 are normal, borderline, and outside normal zones. As an accumulative percentage, a large percentage of study population was found to fall beyond the normal range.

### 3.9. Details of Each Anaemia with respect to Age, Sex, Haemoglobin, and RNFL Thickness

Thalassaemia (beta major + E-beta) was found in 99 patients, of which 69 female and 30 male. The range of haemoglobin was from 3.6 gm/dl to 11 gm/dl with a mean value being 6.9 gm/dl. Of the total 208 eyes, 66 eyes were outside normal limit, 27 eyes were in borderline, and 105 eyes were in normal limit. Of the 99 patients, the mean value of the RNFL of the right eye nasal quadrant was 93.15 *μ*m, temporal quadrant was 75.30 *μ*m, superior nasal quadrant was 120 *μ*m, superotemporal quadrant was 117 *μ*m, inferonasal quadrant was 116 *μ*m, and inferotemporal was 137.6 *μ*m. The left eye values have been found as follows: nasal quadrant 82.2 *μ*m, temporal quadrant 74.24 *μ*m, superior (nasal and temporal) quadrants 120 *μ*m and 117.5 *μ*m, and inferior (temporal and nasal) quadrants 113 *μ*m and 129 *μ*m, respectively.

Iron deficiency anaemia was found in 33 patients, of which 21 were female and 12 were male. The range of haemoglobin varied from 7 gm/dl to 10.5 gm/dl with the mean value being 8.2 gm/dl; out of the total 33 patients, that is, 66 eyes, right eye distributions are as follows: nasal and temporal quadrants are 111.1 *μ*m and 81.01 *μ*m, respectively. For the superior (nasal and temporal) quadrants, the values are 136.1 *μ*m and 142.1 *μ*m, respectively. For the inferior (nasal and temporal) quadrants, the values are 122.1 *μ*m and 101.1 *μ*m, respectively. In the left eye distribution of RNFL thickness, values are 89.09 *μ*m and 75.53 *μ*m for nasal and temporal zones and 113.1 *μ*m and 92.1 *μ*m for superior (nasal and temporal) zones, respectively. For inferior (nasal and temporal) zones, the values are 134.9 *μ*m and 133.9 *μ*m, respectively.

Chronic anaemia: the total number of patients were 18 (36 eyes), of which 6 are female and 12 are male. The range of haemoglobin is 7–10 gm/dl, with mean being 7.07 gm/dl. The mean RNFL thickness values are 109.11 *μ*m and 85.5 *μ*m for nasal and temporal quadrants. For superior (nasal and temporal) quadrants, the values are 153 *μ*m and 124.1 *μ*m. For inferior (nasal and temporal) quadrants, the values are 133.1 *μ*m and 109.1 *μ*m, respectively. For the left eye, the mean value in nasal and temporal quadrants is 81.1 *μ*m and 91.1 *μ*m, respectively, for superior (nasal and temporal) quadrants are 125.1 *μ*m and 76.5 *μ*m, and for inferior (nasal and temporal) quadrants are 117.3 *μ*m and 137.1 *μ*m, respectively.

Tables 3 and 4 show distribution of RNFL in each quadrant and in combination.

**Figure 3:**
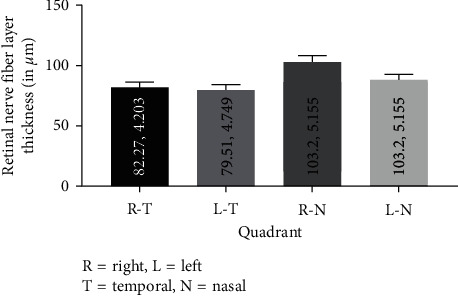
The distribution of the RNFL in each quadrant wise. Retinal nerve fibre layer thickness for each zone (mean, standard error of mean).

Figure 3 shows maximum thinning of RNFL in the nasal quadrant compared to the temporal region.

Figure 2 shows distribution of RNFL thickness altogether in each quadrant in the bar diagram.

### 3.10. Retinal Nerve Fibre Layer (RNFL) Thickness and Haemoglobin Level Correlation

**Figure 4:**
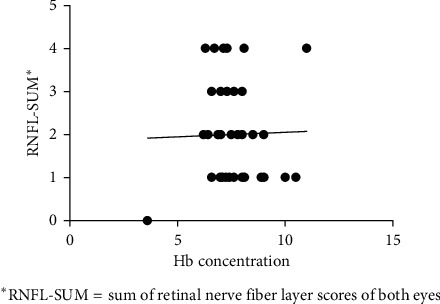
Scatter diagram showing retinal nerve fibre layer (RNFL) thickness and haemoglobin level correlation. Fit plot for the retinal nerve fibre layer vs. haemoglobin concentration.

Figure 4 is a scatter diagram depicting the association between haemoglobin concentration and RNFL thickness. It is clearly shown that almost all data of haemoglobin fall between 5 gm/dl and 10 gm/dl (*X* axis). *Y* axis shows the distribution of RNFL thickness in both eyes as sum total. Most of the abnormal values are shown to be gathering around between 5 gm/dl and 10 gm/dl haemoglobin value.

Stata v14.0 (Stata Corps Inc.) was used to find the relationship between RNFL thinning and “the dependent factors.” Initially, each eye was separately assigned a value (0, 1, and 2) as per the severity of thinning, and then, the sum of the scores of both eyes was considered as a separate variable, and a multiple linear regression analysis was performed with the independent variables being age, sex, Hb level, years of transfusion, and type of anaemia. Once an association was found, we performed a multiple multivariate regression analysis to check the effect of the above independent factors on specific quadrants of each eye and find out a predictive value for such changes. Graph pad prism and stata were used to plot the graphs.

**Figure 5:**
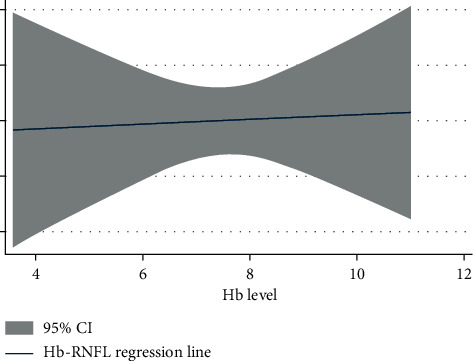
Correlation between the Hb level and RNFL thickness. Fit plot for the RNFL vs. Hb regression analysis.

Figure 5 is the graph showing the correlation between the haemoglobin value and RNFL thinness. Maximum thinness of RNFL (“*y*” axis) was found to be correlating with the haemoglobin value around 7-8 gm/dl (“*x*” axis). The *P* value for this correlation was found to be 0.02, which is statistically significant.

## 4. Discussion

This study is the first kind of study from eastern India to analyse RNFL thickness in three different categories of population.

### 4.1. Age Distribution

Mean age of this study population was much higher (23.6 years) compared to other studies like by Uzun et al. [13] where the mean age of the study population was much younger (average 13.7 years). This study was targeted not only to thalassaemia but also to iron deficiency and chronic anaemia, which included larger number of adult population.

### 4.2. Gender Distribution

Sex distribution among the study population was found to corroborate with various previous studies [13–15].

### 4.3. Haemoglobin Level

The mean value of haemoglobin in this study was found to be 7.5 gm/dl. This indicates a larger burden of anaemic hypoxia in large number of patients. It also imposed more number of required blood transfusion in those population. These findings are almost same with other studies performed previously.

### 4.4. Blood  Transfusion and Serum Ferritin

Blood transfusion distribution in thalassaemia was studied to study the relationship of iron overload and RNFL thinning as a consequence of recurrent transfusions and ineffective erythropoiesis. Although iron is crucial for the synthesis of neurotransmitters, optic nerve myelination, and visual phototransduction cascade, excessive iron may be toxic. Iron has a catalytic role to produce reactive oxygen species and free radicals, which may lead to oxidative damage. Oxidative damages caused by free radicals include DNA strand breakage, lipid peroxidation, and biomolecule; it has been known that the retina and RPE are particularly prone to oxidative damage caused by iron overload. This study showed increased RNFL thinning in anaemic, thalassaemic patients who had received more number of transfusion. Although no statistical significance was found, a numerically higher value was obtained. Almost all patients were on iron chelation therapy, and the mean serum ferritin level depicted is given in Table 3. Therefore, the serum ferritin level may not be a good predictor of RNFL thinning in patients receiving oral chelation therapy and taking blood transfusions for long.

### 4.5. Retinal Nerve Fibre Correlation between Our Study Group and Normal Human Population

In this study, thinning of the RNFL was found in patients suffering from anaemia, thalassaemia. All quadrants showed thinning in the RNFL as measured by OCT. Compared to the normal value obtained in various previous studies, these values were much lower and below two standard deviations [16].

Maximum thinning was obtained in the nasal quadrant compared to other quadrants. In the decreasing order of thinning, the most affected is the nasal quadrant, followed by temporal, inferior, and superior quadrants.

### 4.6. Haemoglobin and RNFL Correlation

Correlation between the Hb level and RNFL thickness was analysed in this study. A proper statistical method was applied. Initially, the RNFL thickness was analysed and then compared as cumulative percentage. Linear regression and multivariate regression formulae were applied. To find the correlation and regression values, the Cox linear model and Beau multivariate model were applied.

It was found that the thickness of RNFL showed a decreasing trend along with the decrease in the Hb level. It showed a positive correlation with the *P* value of 0.02 (statistically significant) and with a 95% confidence interval falling in between significant values. The maximum thinning was found at the Hb level of 7.6 gm/dl. The mean Hb level in this study was 7.5 gm/dl. Therefore, it is worthwhile to mention that the more anaemic a person, the more thinning of the RNFL might occur. Therefore, anaemia is a strong potential indicator of RNFL thinning as evident from this study.

In this study, 64% of patients was thalassaemic, 22% was iron deficient anaemic, and 12% was suffering from anaemia of chronic disease. No previous studies till now had included such categorical patients neither they had found any significant association between the haemoglobin level and RNFL thinning, although they showed loss of the RNFL in all quadrants in such patients.

## 5. Conclusion

Thalassaemia syndrome, which is a common problem, poses a huge burden in Indian population. RNFL thickness in these patients decreases with the decrease in haemoglobin concentration as evident in this study. Moreover, this study established a statistical significance between two of them.

Iron deficiency anaemia (IDA) is the most prevalent anaemia worldwide. The prevalence of IDA is estimated to be quite high in Indian population. RNFL thickness was found to be correlating positively with the decreasing haemoglobin level.

Anaemia of chronic diseases (ACD) is associated with several illness. ACD also impacts RNFL like thalassaemia and IDA as evident from this study.

OCT was found to be an important diagnostic measure, which can help in early detection of RNFL thinning. Therefore, measurements of RNFL by OCT in anaemic diseases spectrum might help in detection of early complication of such potential dangerous impact and will definitely help in starting effective strategies in such grave circumstances.

## Figures and Tables

**Table 1:** Value of haemoglobin (Hb) and RBC indices in different subsets of anaemic population.

Category (*n*)	Hb (gm/dl)	MCV (fl)	MCH (pg)	MCHC (gm/dl)
Thalassaemia (99)	6.9	62	24.7	30.6
IDA (33)	8.2	67	26.3	31.4
Chronic anaemia (18)	7.07	84	32	35.6

**Table 2:** Serum ferritin value in different subsets of anaemic population.

Category (number of patients)	Serum ferritin (ng/ml) (range/mean value)
Thalassaemia (99)	995–4369 (2438)
Iron deficiency anaemia (33)	14–18.3 (16.4)
Anaemia of chronic disease (18)	46–79 (67)

**Table 3:** Distribution of mean RNFL (in micrometres) in nasal, temporal, superior, and inferior quadrants in the study population.

	Nasal	Temporal	Superior	Inferior
Thalassaemia (*n* = 99)				
Right eye	93.15	75.30	118.75	126.5
Left eye	82.2	74.24	126	121.2

Iron deficiency anaemia (*n* = 33)				
Right eye	111.11	81.01	139.25	112.5
Left eye	89.01	75.53	102.5	133.5

Anaemia of chronic disease (*n* = 18)				
Right eye	109.11	85.5	138.65	121
Left eye	91.1	88.11	100.5	127

**Table 4:** Distribution of mean RNFL (in micrometres) for each quadrant of the eye in the study population.

	Superonasal	Superotemporal	Inferonasal	Inferotemporal
Thalassaemia (*n* = 99)				
Right eye	120	117.5	116	137.6
Left eye	115	137.7	113.1	129.1

Iron deficiency anaemia (*n* = 33)				
Right eye	136.1	142.1	122.1	100.1
Left eye	113.3	92.1	134.9	133.3

Anaemia of chronic disease (*n* = 18)				
Right eye	153	124.1	133.1	109.1
Left eye	125.1	76.5	117.3	137.1

## Data Availability

The detailed data type used to support the findings of this study is available from the corresponding author upon request.

## References

[1] Walia S., Fishman G. A., Edward D. P., Lindeman M. (2007). Retinal nerve fiber layer defects in RP patients. *Investigative Opthalmology & Visual Science*.

[2] Hoyt W. F., Schlicke B., Eckelhoff R. J. (1972). Fundoscopic appearance of a nerve-fibre-bundle defect. *British Journal of Ophthalmology*.

[3] Kaur C., Foulds W. S., Ling E. A. (2008). Hypoxia-ischemia and retinal ganglion cell damage. *Clinical Ophthalmology*.

[4] Leung C. K. S., Tham C. C. Y., Mohammed S. (2007). In vivo measurements of macular and nerve fibre layer thickness in retinal arterial occlusion. *Eye (London, England)*.

[5] Kergoat H. L. N., Hérard M.-E. V., Lemay M. (2006). RGC sensitivity to mild systemic hypoxia. *Investigative Opthalmology & Visual Science*.

[6] Stedman T. L. (2006). *Stedman’s Medical Dictionary*.

[7] GBD 2015 Disease and Injury Incidence and Prevalence Collaborators (2016). Global, regional, and national incidence, prevalence, and years lived with disability for 310 diseases and injuries, 1990–2015: a systematic analysis for the global burden of disease study 2015. *Lancet*.

[8] Olivieri O., De Franceschi L., Capellini M. D., Girelli D., Corrocher R., Brugnara C. (1994). Oxidative damage and erythrocyte membrane transport abnormalities in thalassemias. *Blood*.

[9] Cohen-Cory S., Lom B. (2004). Neurotrophic regulation of retinal ganglion cell synaptic connectivity: from axons and dendrites to synapses. *The International Journal of Developmental Biology*.

[10] NHLBI (2012). *What are the Signs and Symptoms of Thalassemias?*.

[11] Kah T. (2018). CuRRL syndrome: a case series. *Acta Scientific Ophthalmology*.

[12] De Carlo T. E., Romano A., Waheed N. K., Duker J. S., Duker Jay S. (2015). A review of optical coherence tomography angiography (OCTA). *International Journal of Retina and Vitreous*.

[13] Uzun F., Karaca E. E., Yıldız Yerlikaya G. (2017). Retinal nerve fiber layer thickness in children with *β*- thalassemia majorSaudi. *Saudi Journal of Ophthalmology*.

[14] Akdogan E., Turkyilmaz K., Ayaz T., Tufekci D. (2015). Peripapillary retinal nerve fibre layer thickness in women with iron deficiency anaemia. *Journal of International Medical Research*.

[15] Cikmazkara I., Ugurlu S. K. (2016). Peripapillary retinal nerve fiber layer thickness in patients with iron deficiency anemia. *Indian Journal of Ophthalmology*.

[16] Varma R., Skaf M., Barron E. (1996). Retinal nerve fiber layer thickness in normal human eyes. *Ophthalmology*.

